# Deciding Without Intending

**DOI:** 10.5334/joc.101

**Published:** 2020-06-02

**Authors:** Alexandra Nolte, Wesley Buckwalter, David Rose, John Turri

**Affiliations:** 1 Department of Philosophy, Florida State University, US; 2 Department of Philosophy, University of Manchester, GB; 3 Philosophy Department and Cognitive Science Program, University of Waterloo, CA

**Keywords:** decision, intention, judgment, meaning, semantics, ordinary language

## Abstract

According to a consensus view in philosophy, “deciding” and “intending” are synonymous expressions. Researchers have recently challenged this view with the discovery of a counterexample in which ordinary speakers attribute deciding without intending. The aim of this paper is to investigate the strengths and limits of this discovery. The result of this investigation revealed that the evidence challenging the consensus view is strong. We replicate the initial finding against consensus and extend it by utilizing several new measures, materials, and procedures. Together this evidence strongly suggests that “deciding” is not synonymous with “intending” in ordinary language and that the consensus view should be rejected.

## 1. Introduction

If someone decides to do something, do they thereby intend to do it? According to a popular view in action theory and philosophy of mind, the answer is “yes” (McCann 1986; Frankfurt 1988; Kane 1998; Searle 2001; Clarke 2003; Mele 2003; Carruthers 2010; Kaufman 1966; Hall 1978; Pink 1996; Holton 2009; Shepherd 2013; Goldman 1970; Meiland 1970; Raz 1975; Aune 1978; O’Shaughnessy, 1980). Call this view about the relationship between decisions and intentions the *equivalence thesis*. According to the equivalence thesis, “deciding to X” and “forming an intention to X” are synonymous expressions. So, for example, if a chairman of a company decided to increase the company’s CO_2_ emissions, then he must have also formed an intention to increase the emissions. And similarly, if the chairman formed an intention to increase the company’s CO_2_ emissions, then he also must have decided to do that. The equivalence thesis is often represented as the “received view” that has generated “virtually no debate” about the semantic connection between “deciding” and intending” (Aune 1978, p. 564; McGuire 2016, p. 270).

Against the received view, it has recently been argued that the equivalence thesis is false (McGuire 2016). If “deciding” and “forming an intention” to do something really are synonymous, one implication of this is that whenever a person decides to do something that person also intends to do it.^1^ However researchers have discovered a case in which it is natural to say that someone has decided to do something but has not formed an intention to do it (McGuire 2016). In this case, a significant number of ordinary English speakers attribute deciding without intending. The experiment is as follows. Participants were presented with the following stimulus:

The vice-president of a company went to the chairman of the board and said, ‘We are thinking of starting a new program. It will help us increase profits, but it will also increase our CO_2_ emissions’. The chairman had mixed feelings about this. He wanted to increase the company’s profits, but he did not want to increase its CO_2_ emissions. The company had a good reputation for environmental responsibility and the chairman did not want to change that. After carefully considering the matter, the chairman instructed the vice-president to start the new program. They started the program. Sure enough, the company’s profits increased and so did its CO_2_ emissions.

After seeing this case, one group of participants was asked to what extent they agreed that “the chairman decided to increase the company’s CO_2_ emissions,” while another group of participants was asked to what extent they agreed with “the chairman intended to increase the company’s CO_2_ emissions.” The results indicated that participants in the former group were more inclined to agree that the chairman decided to increase the emissions than the participants in the latter group were to agree that the chairman intended to increase the emissions. McGuire concluded that ordinary judgments observed in response to the case above strongly suggest that the equivalence thesis is false.

As the received view in philosophy, it would be surprising if the equivalence thesis turned out to be false. The experimental evidence against the equivalence thesis is also limited in various respects. For example, the evidence consists of a single experiment utilizing a single measure and narrative case. It is well known that multiple replication attempts involving a wide range of participants, measures, and materials can increase the likelihood that initial discoveries are reliable and generalizable (Open Science Collaboration 2015; see also Buckwalter, 2019; Buckwalter & Turri, 2018; Rose, Buckwalter & Nichols, 2017; Rose, Machery, Stich et at., 2019; Turri, 2017; Turri, 2018; Machery & Doris, 2017). Thus, it is reasonable to investigate this discovery in further detail before drawing strong theoretical conclusions.

The following experiments were planned, pre-registered, and conducted with precisely this goal in mind. With respect to participants, prior research (e.g. McGuire 2016) has challenged the equivalence thesis by probing one group of participants about “deciding”, another group about “intending”. This approach leaves open the question of whether the same participants judge that one can decide to something without intending to do it. Experiment 1 successfully replicates the original discovery and demonstrates that the same participant is more inclined to attribute deciding than intending. With respect to measures, prior research (e.g. McGuire 2016) has challenged the equivalence thesis by probing participants in one particular way. This leaves open the question that the results are an artifact of the question procedures. Experiments 2–4 replicate the original discovery—sometimes successfully, sometimes unsuccessfully—by probing deciding and intention judgments in multiple ways. With respect to materials, McGuire (2016) has challenged the equivalence thesis through a single empirically substantiated counterexample. This leaves open the possibility that the pattern of findings was due to incidental features of the case that do not generalize. Experiment 5 successfully replicates and generalizes the original finding with several new materials. Across a wide range of cases, people are overwhelmingly inclined to attribute deciding without attributing the formation of an intention. Expanding on the original discovery in each of these ways makes a powerful case that “deciding” is not synonymous with “intending” and advances our understanding of the strengths and weaknesses of the evidence for this claim.

## 2. General Methods

The following statements are true of all studies reported here. All manipulations, measures, and exclusion criteria are reported. All participants were adult residents of the United States. We recruited and tested people using an online platform of Amazon Mechanical Turk (https://www.mturk.com), TurkPrime (Litman, Robinson, and Abberbock 2017), and Qualtrics (https://www.qualtrics.com). Participants completed a brief demographic questionnaire after testing. We used R 3.5.2 for all analyses (R Core Team 2018). All stimuli and data are available through an Open Science Foundation project (osf.io/m94zg). All studies were pre-registered.

## 3. Experiment 1

### 3.1. Method, Participants, and Procedure

Following McGuire (2016), we decided in advance to recruit 100 participants per condition, plus some extra as a precaution against attrition (see pre-registration). Two hundred nine people participated in the study. Their mean age was 35.27 years (range = 20–70, sd = 10.35), 41% (86 of 209) were female, and 94% reported native competence in English.

Participants were randomly assigned to one of two conditions that differed in the order of test statements (decision-first, intend-first). Participants read a brief scenario taken verbatim from previous research printed in the introduction (McGuire 2016; see also Knobe 2003). Participants then responded to the first test statement on the same screen, went to a new screen and completed a distractor task consisting of responding to four attributions, then went to a new screen and responded to second test statement. The test statements were taken verbatim from previous research (McGuire 2016).

- Test statements:
  –The chairman intended to increase the company’s CO_2_ emissions. (intend)
  –The chairman decided to increase the company’s CO_2_ emissions. (decide)
- Distractor statements:
  –The chairman hoped to increase the company’s CO_2_ emissions.
  –The chairman cared about increasing the company’s CO_2_ emissions.
  –The chairman was excited about increasing the company’s CO_2_ emissions.
  –The chairman was happy to increase the company’s CO_2_ emissions.

Responses were collected on the same 7-point Likert scale used in previous research (McGuire 2016), anchored with “1: disagree”, “4: neither agree nor disagree”, and “7: agree”. The options appeared vertically on smaller screens and left-to-right on larger screens capable of displaying the options horizontally.

### 3.2. Results

Our principal research question was whether decision attributions would exceed intent attributions. To answer this question, we conducted a linear mixed effects analysis on participant response and followed up with appropriate t-tests. The model included order, judgment type (within-subjects: decide, intend), and participant age and sex as fixed effects. It also included a random intercept for participant.

**Figure 1:**
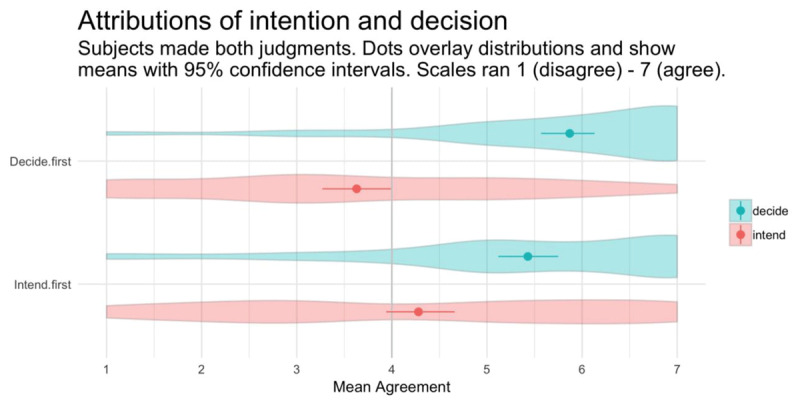
Experiment 1. Mean response overlaying distributions for decision and intent attributions (within-subjects) across two statement orders (between-subjects). Scales ran 1 (“disagree”)–7 (“agree”). Error bars show 95% bootstrapped confidence intervals.

The linear mixed effects analysis revealed a main effect of judgment on participant response, qualified by an interaction between order and judgment (see Table 1 and Figure 1). Follow-up paired samples t-tests revealed that the mean decision attribution was significantly higher in both order conditions, but the effect size was larger in the decide-first condition (see Table 2). Mean decision attribution was significantly above the midpoint in both order conditions. Mean intent attribution was non-significantly above the midpoint in the intend-first condition, and it was significantly below the midpoint in the decide-first condition (see Table 3).

**Table 1:** Experiment 1. Analysis of variance for the linear mixed model’s fixed effects.

	Sum of squares	Df1	Df2	F	p

Order	0.644	1	205	0.267	.606
Judgment	299.107	1	207	124.092	<.001
Sex	2.061	1	205	0.855	.356
Age	0.001	1	205	0.000	.985
Order:Judgment	31.164	1	207	12.929	<.000

**Table 2:** Experiment 1. Paired samples t-tests for decision and intent attributions in the two order conditions.

Order	estimate	95 CI low	95 CI high	df	t	p	d

Intend first	1.147	0.738	1.556	108	5.555	<.001	0.532
Decide first	2.240	1.796	2.684	99	10.005	<.001	1.001

**Table 3:** Experiment 1. Descriptive statistics for decision and intent attributions in the two order conditions, along with the results of one sample t-tests against the neutral midpoint (test-value = 4).

Order	Judgment	n	median	SD	mean	95 CI low	95 CI high	df	t	p	d

Intend first	intend	109	5	2.01	4.28	3.90	4.66	108	1.47	.14	0.14
Intend first	decide	109	6	1.65	5.43	5.09	5.75	108	9.04	<.001	0.87
Decide first	intend	100	3	1.81	3.63	3.26	4.00	99	–2.05	.04	–0.20
Decide first	decide	100	6	1.47	5.87	5.57	6.16	99	12.74	<.001	1.27

### 3.3. Discussion

This study was a pre-registered attempt to replicate the discovery that people judged that an agent decided to do something despite not judging that he intended to do it. The finding replicated. The original finding was observed entirely between-subjects, leaving open the possibility that the same group of participants would not attribute a decision without also attributing an intention. The present study adopted a mixed design, treating judgment as a within-subjects factor and the order of judgments as a between-subjects factor, allowing us to accomplish two things at once. On the one hand, by comparing intent attributions in the intend-first condition to decision attributions in the decide-first condition, we achieved an exact replication of the original between-subjects finding. On the other hand, by having all participants record both judgments and manipulating judgment order, we extended the finding in two informative ways. First, we showed that the same participants tend to attribute a decision without also attributing a corresponding intention. Second, this occurred regardless of the order in which the attributions occurred.

## 4. Experiment 2

One concern about the findings from Experiment 1 is that they could have been an artifact of the stimuli and procedures. The scenario describes a stereotypical “decision-making” context, so participants who rated only a single scaled item might have tended to agree in order to indicate their awareness that the chairman had made a salient decision. This could lead participants to agree that the chairman decided to harm the environment even though they were mainly interested in conveying that he decided to *start the program*, which he knew would harm the environment. One motivation for answering this way could be to express willingness to hold the chairman accountable for foreseeable negative consequences of his decisions. By contrast, the scenario does not describe a stereotypical “intention-making” context — such a phrase has no currency in contemporary American English — so participants would not be similarly motivated to agree with a single scaled item about intention. In order to address this set of issues, the present study used a different questioning procedure that explicitly distinguished *deciding to harm the environment*, on the one hand, from *knowing that a decision would harm the environment*, on the other. If participants continue counting the chairman as having decided to harm the environment, then it would address the concern and strengthen the principal finding from Experiment 1. By contrast, if participants no longer count the chairman as having made that decision, then it validates the concern and undermines the earlier finding.

### 4.1. Method, Participants, and Procedure

We decided in advance to recruit 100 participants plus some extra as a precaution against attrition (see pre-registration). One hundred four people participated in the study. Their mean age was 36.03 years (range = 18–70, sd = 9.88), 50% (52 of 104) were female, and 92% reported native competence in English.

Participants read the same scenario from Experiment 1, responded to two test items beneath the text of the scenario, then went to a new screen and responded to a comprehension question, “Did CO_2_ emissions increase when the company started the new program?” and responded by indicating “yes” or “no”. Each test item began with the question, “Which better describes the chairman?” Options for the intent attribution were:

- He intended to start the program and he knew that this would cause environmental damage.
- He intended to start the program and he intended to cause environmental damage.

And options for the decision attribution were:

- He decided to start the program and he knew that this would cause environmental damage.
- He decided to start the program and he decided to cause environmental damage.

The order of the test statements and the response options was rotated randomly.

### 4.2. Results

For each test item, we counted the option with “he knew” as a denial (coded “0”) and we counted the other option as an attribution (coded “1”). Our principal research question was whether decision attributions would exceed intent attributions with respect to environmental damage. To answer this question, we conducted a generalized linear mixed-effects analysis followed up with appropriate proportion tests. The model included judgment type (within-subjects: decide, intend) and participant age and sex as fixed effects. It also included a random intercept for participant.

**Figure 2:**
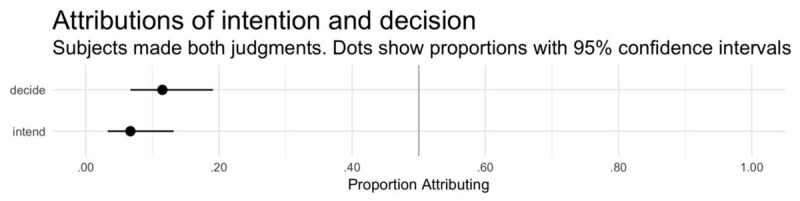
Experiment 2. Proportion of participants attributing intent and decision (within-subjects). Error bars show 95% bootstrapped confidence intervals.

The vast majority of participants (101 of 104) correctly answered the comprehension question, indicating that they understood the scenario’s details. All participants are included in the analyses below. The generalized linear model revealed a main effect of judgment type (see Figure 2 and Table 4). However, the model suffered from quasi-complete separation, with only one participant selecting one of the four combinations of answers to the two test items (deny intent + attribute decision; see Table 6), and a follow-up McNemar’s test was insignificant, χ^2^(1) = 2.29, p = .131, h = –0.168. Follow-up binomial tests revealed that each attribution was significantly below chance rates (see Table 5). The vast majority of participants (~88%) denied both statuses (see Table 6).

**Table 4:** Experiment 2. Generalized linear model’s fixed effects.

term	estimate	std. error	z	p

(Intercept)	–17.270	6.433	–2.685	.007
Decide	7.577	2.463	3.076	.002
Female	0.148	2.716	0.054	.957
Age	–0.033	0.152	–0.216	.829

**Table 5:** Experiment 2. Descriptive statistics, binomial tests, and effect sizes for the two attributions.

Judgment	n	k	prop	95 CI low	95 CI high	test value	p	h

intend	104	7	.067	.033	.132	.5	<.001	–1.046
decide	104	12	.115	.067	.191	.5	<.001	–0.878

**Table 6:** Experiment 2. Counts of participants who made the four possible combinations of judgments (intent × decision: deny (= 0) or attribute (= 1)).

Intend	Decide	Frequency

0	0	91
1	0	1
0	1	6
1	1	6

### 4.3. Discussion

This experiment attempted to replicate the principal finding from Experiment 1 while addressing a concern that decision attributions were inflated due to the stimuli and questioning procedures. To address the concern, the present experiment used a different questioning procedure that explicitly distinguished deciding to harm the environment, on the one hand, from knowing that another decision would cause harm to the environment, on the other. When questioned this way, participants no longer judged that the chairman decided to harm the environment. Instead, they preferred the description that he knew that his decision to start the program would harm the environment. Moreover, they attributed intent and decision at similar, very low rates. This suggests that the earlier finding was an artifact and does not clearly support the conclusion that, on the ordinary view, one can decide to something without forming an intention to do it.

## 5. Experiment 3

One concern about the findings from Experiment 2 is that they might not be informative regarding the principal research question in the following sense. Suppose that when asked to select which option better describes the chairman, participants tend to prefer “he knows that it will cause damage” to “he decided/intended to cause damage.” This is consistent with participants thinking that both descriptions are true, even if one is judged to be better for some reason. In order to address this concern, the present experiment used a different questioning procedure. Instead of asking participants to select the better description, we asked them to select all the descriptions that applied (Guglielmo and Malle 2010; though see Cova, Lantian & Boudesseul 2016). If participants continue counting the chairman as having decided to increase emissions without intending to do so, then it would strengthen the principal finding from Experiment 1. By contrast, if participants don’t do that, then it undermines the finding from Experiment 1.

### 5.1. Method, Participants, and Procedure

We decided in advance to recruit 100 participants plus some extra as a precaution against attrition (see pre-registration). One hundred six people participated in the study. Their mean age was 34.81 years (range = 19–67, sd = 10.81), 38% (40 of 106) were female, and 95% reported native competence in English.

Participants read the same scenario from Experiment 1, responded to six test items beneath the text of the scenario (see list below), then went to a new screen and responded to a the same comprehension question used in experiment 2. Preceding the test items were the instructions, “Please select all that apply to the chairman.” The following list appeared in randomized order:

- He decided to increase emissions.
- He intended to increase emissions.
- He is responsible for increasing emissions.
- He wanted to increase emissions.
- He knowingly increased emissions.
- He was hired to increase emissions.

Participants selected an item by tapping or clicking on the relevant textbox. Text boxes were light gray with dark gray font. Selecting a textbox highlighted it blue and turned its text white. Items could be unselected by tapping or clicking again, which returned the textbox to light gray with dark gray font.

### 5.2. Results

For each test item, we coded it “1” if it was selected and “0” if it wasn’t. Our principal research question was whether decision attributions would exceed intent attributions. To answer this question, we conducted a generalized linear mixed-effects analysis followed up with appropriate proportion tests. The model included judgment type (within-subjects: decide, intend) and participant age and sex as fixed effects. It also included a random intercept for participant.

**Figure 3:**
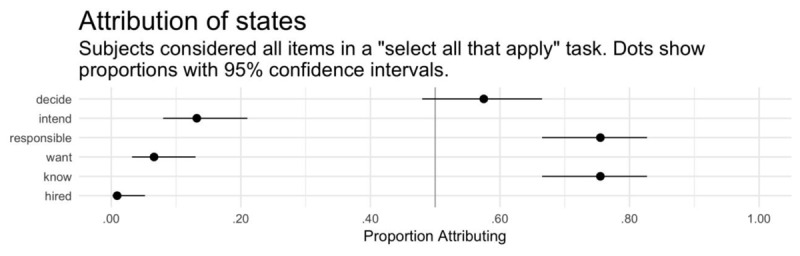
Experiment 3. Proportion of participants attributing intent and decision (within-subjects). Error bars show 95% bootstrapped confidence intervals.

The vast majority of participants (101 of 106) correctly answered the comprehension question, indicating that they understood the scenario’s details. All participants are included in the analyses below. The generalized linear model revealed a main effect of judgment type (see Figure 3 and Table 7), with participants significantly more likely to attribute decision than intention. A follow-up McNemar’s test was significant, χ^2^(1) = 41.49, p < .001, h = –0.978. Follow-up binomial tests revealed that intent attribution was significantly below chance rates, whereas decision attribution was non-significantly above chance rates (see Table 8). Nearly half of participants (46.22%) attributed decision but denied intent (see Table 9), which far exceeds chance rates, binomial test, k = 49, n = 106, p < .001.

**Table 7:** Experiment 3. Generalized linear model’s fixed effects.

term	estimate	std. error	z	p.value

(Intercept)	–3.647	1.021	–3.571	<.001
Decide	2.895	0.630	4.598	<.001
Female	0.341	0.466	0.730	.465
Age	0.030	0.021	1.436	.151

**Table 8:** Experiment 3. Descriptive statistics, binomial tests, and effect sizes for the test items.

Judgment	n	k	prop	95 CI low	95 CI high	test value	p	h

decide	106	61	.575	.480	.665	.5	.145	0.152
intend	106	14	.132	.080	.210	.5	<.001	–0.827
responsible	106	80	.755	.665	.827	.5	<.001	0.535
want	106	7	.066	.032	.130	.5	<.001	–1.051
know	106	80	.755	.665	.827	.5	<.001	0.535
hired	106	1	.009	.002	.052	.5	<.001	–1.376

**Table 9:** Experiment 3. Counts of participants who made the four possible combinations of intent and decision: deny (= 0) or attribute (= 1).

Intend	Decide	Frequency

0	0	43
1	0	2
0	1	49
1	1	12

### 5.3. Discussion

This study attempted to replicate the principal finding from Experiment 1 using a select-all-that-apply task. When questioned this way, participants continued to attribute a decision to increase emissions without attributing a corresponding intention to do so. This supports the original finding and strengthens the conclusion that, on the ordinary view, one can decide to do something without forming an intention to do it.

## 6. Experiment 4

One concern about the findings from Experiment 3 is that the questioning procedures eliminated the contrast between starting the program and increasing emissions. As discussed above, the scenario describes a stereotypical “decision-making” context, so when offered only one opportunity to rate a decision attribution, participants might have agreed in order to indicate their awareness that the chairman had made a salient decision. Participants might have answered that the chairman decided to increase emissions even though they were mainly interested in conveying that he decided to start the program, or that he was responsible for increasing emissions. But the scenario does not describe a stereotypical “intention-making” context, so the same considerations don’t apply to that attribution. In order to address this, the present study gave participants the opportunity to rate, in the same context, attributions for both starting the program and increasing emissions. If participants continue counting the chairman as having decided to increase emissions without intending to do so, then it would strengthen the principal finding from Experiment 1 and 3. By contrast, if participants don’t do that, then it undermines the finding from Experiment 1 and 3.

### 6.1. Method, Participants, and Procedure

We decided in advance to recruit 100 participants plus some extra as a precaution against attrition (see pre-registration). One hundred five people participated in the study. Their mean age was 37.68 years (range = 21–72, sd = 11.78), 36% (38 of 105) were female, and 93% reported native competence in English.

Participants read the same scenario from Experiment 1, responded to three test items beneath the text of the scenario, then went to a new screen and responded to the same comprehension question used in the previous experiments. Preceding the test items were the instructions, “In what follows, please select all that apply. For each question, you can select both options, either one separately, or neither.” The following list appeared in randomized order, with response options appearing in random order horizontally below the question:

- What did the chairman decide to do? [start the program/increase emissions]
- What did the chairman intend to do? [start the program/increase emissions]
- What is the chairman responsible for? [starting the program/increasing emissions]

Participants selected items in the same way as in Experiment 3.

### 6.2. Results

For each test item, we coded participant response as “1” if it was selected and “0” if it wasn’t. Our principal research question was whether decision attributions would exceed intent attributions. To answer this question, we conducted a generalized linear mixed-effects analysis followed up with appropriate proportion tests. The model included judgment type (within-subjects: decide, intend), focus (within-subjects: program, emissions), and participant age and sex as fixed effects. It also included a random intercept for participant.

**Figure 4:**
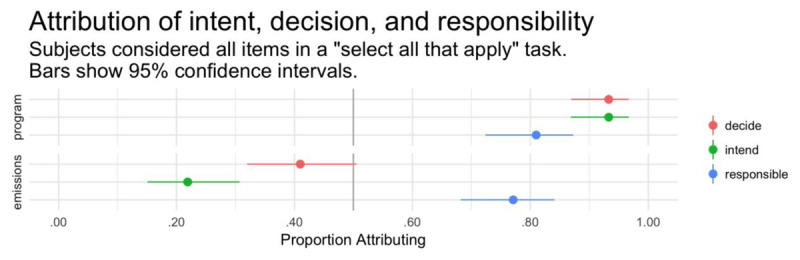
Experiment 4. Proportion of participants attributing intent, decision, and responsibility (within-subjects). Error bars show 95% bootstrapped confidence intervals.

The vast majority of participants (97 of 105) correctly answered the comprehension question, indicating that they understood the scenario’s details. Again, all participants were included for data analyses. The generalized linear model revealed a main effect of focus (see Figure 4 and Table 10), with participants significantly less likely to make an attribution for increasing emissions than for starting the program. There was no effect of judgment and no interaction between judgment and focus. However, a follow-up McNemar’s test directly comparing decision and intent attributions for emissions was significant, χ^2^(1) = 12.89, p < .001, h = –0.415. Follow-up binomial tests revealed that intent and decision attributions for starting the program were significantly above chance rates; by contrast, for increasing emissions, intent attributions were significantly below chance rates and decision attributions were trending below chance rates (see Table 11). Less than one quarter of participants (24 of 105) attributed decision but denied intent, which does not differ from chance rates, binomial test, k = 24, n = 105, p = .654, test proportion = 0.25 (see Table 12). A statistically significant majority (58 of 105) denied both statuses, binomial test, k = 58, n = 105, p < .001, test proportion = 0.25.

**Table 10:** Experiment 4. Generalized linear model’s fixed effects.

Term	estimate	std. error	z	p.value

(Intercept)	2.006	0.593	3.382	.001
Decide	<.001	0.556	0.000	1
Emissions	–4.043	0.523	–7.735	<.001
Female	0.097	0.296	0.329	.742
Age	0.018	0.012	1.473	.141
Decide:Emissions	0.942	0.642	1.466	.143

**Table 11:** Experiment 4. Descriptive statistics, binomial tests, and effect sizes for the test items.

Judgment	Focus	n	k	prop	95 CI low	95 CI high	test value	p	h

decide	program	105	98	.933	.869	.967	.5	<.001	1.048
decide	emissions	105	43	.410	.320	.505	.5	.078	–0.182
intend	program	105	98	.933	.869	.967	.5	<.001	1.048
intend	emissions	105	23	.219	.151	.307	.5	<.001	–0.597
responsible	program	105	85	.810	.724	.873	.5	<.001	0.668
responsible	emissions	105	81	.771	.682	.841	.5	<.001	0.574

**Table 12:** Experiment 4. Counts of participants who made the four possible combinations of intent and decision: deny (= 0) or attribute (= 1).

Intend	Decide	Frequency

0	0	58
1	0	4
0	1	24
1	1	19

### 6.3. Discussion

This study attempted to replicate the principal finding from Experiment 1 using a select-all-that-apply task while providing participants with potentially meaningful contrasts for the various attributions of intent and decision. Using these procedures, we observed mixed evidence that neither clearly supports nor clearly undermines the initial finding. In favor of the original finding, a direct comparison of intent and decision attributions for increasing emissions yielded a significant difference in the predicted direction: decision attribution was significantly higher than intent attribution. However, against the original finding, this difference was not significant in a full model controlling for other factors, overall participants tended to deny both decision and intent, and the proportion of participants who attributed decision but denied intent was numerically below chance rates.

At this point, we think that two conclusions are warranted. On the one hand, the findings from our first four studies have strengthened the original result suggesting that there is a detectable central tendency to attribute decision without intent (McGuire 2016). In particular, we have shown that this result occurs within-subjects and is not limited to single-item scaled responses (Studies 1 and 3). On the other hand, some concerns about how to interpret the finding remain. First, it does not show up for some ways of probing for judgments, although there could be an exonerating alternative explanation for this (Experiment 2). Second, for other questioning procedures that are less susceptible to alternative explanations, the predicted difference does not occur in a full model that controls for other factors and participants do not attribute decision without intent at rates exceeding chance (Experiment 4).

A possible explanation for these mixed results is that they are somehow due to incidental features of the scenario under evaluation. As mentioned above, two possibilities are that it describes a stereotypical decision-making context and it invites a preoccupation with moral evaluation. Taken together, these features, and perhaps others, could conspire to inflate some decision attributions without inflating the corresponding intention attribution. This deflationary explanation is consistent with some of the findings from our first four studies. And we doubt that it is possible to satisfactorily address the underlying concerns by continuing to study the same scenario. More generally, it is suboptimal to draw firm conclusions about concepts without investigating judgments about a wide range of narrative contexts that differ in their subject matter and other incidental details.

## 7. Experiment 5

One concern about the previous findings is that they are somehow due to incidental features of the scenario tested, which conspired to inflate decision attribution but not intention attribution. In order to address this, the present study tested a range of scenarios. When accounting for variation due to stimulus selection, if decision attribution significantly exceeds intention attribution, then it will support the main findings from previous studies. By contrast, if decision attribution no longer exceeds intention attribution, then it will undermine the earlier findings.

### 7.1. Method, Participants, and Procedure

We decided in advance to recruit 250 participants plus some extra as a precaution against attrition (see pre-registration). Two hundred sixty-one people participated in the study. Their mean age was 36.15 years (range = 18–83, sd = 11.44), 46% (120 of 261) were female, and 92% reported native competence in English.

Participants were randomly assigned to one of five conditions that varied which scenario they read (general, captain, wolf, sunset, robber). After reading the scenario, participants rated attributions of decision and intention; the order of attributions and response options were rotated randomly. All stimuli are included in this project’s OSF repository (osf.io/m94zg). Below is an example used in one condition.

(Sunset) Maria and Mike are on their way to a work dinner. Mike tells Maria that he is going to take the Parkway so they can see the sunset, as he heard it is going to be one to remember. Maria responds by saying, “But Mike, if you take the Parkway instead of the Interstate we will be late to the dinner.” Mike thinks this over for a moment and responds by saying, “If we could take the Interstate and see the sunset, I would drive that way. However, that is simply impossible because it will be behind us and I’m driving.” Mike takes the Parkway. They see the sunset and are late for the dinner.

Mike decided to arrive late to the dinner. (no/yes)

Mike intended to arrive late to the dinner. (no/yes)

### 7.2. Results

For each test item, we coded “yes” as “1” and “no” as “0”. Our principal research question was whether decision attributions would exceed intent attributions when accounting for the variability due to stimulus selection. To answer this question, we conducted a generalized linear mixed-effects analysis. The model included fixed effects of judgment type (within-subjects: decide, intend) and participant age and sex; it also included random intercepts for scenario and participant nested within scenario.

**Figure 5:**
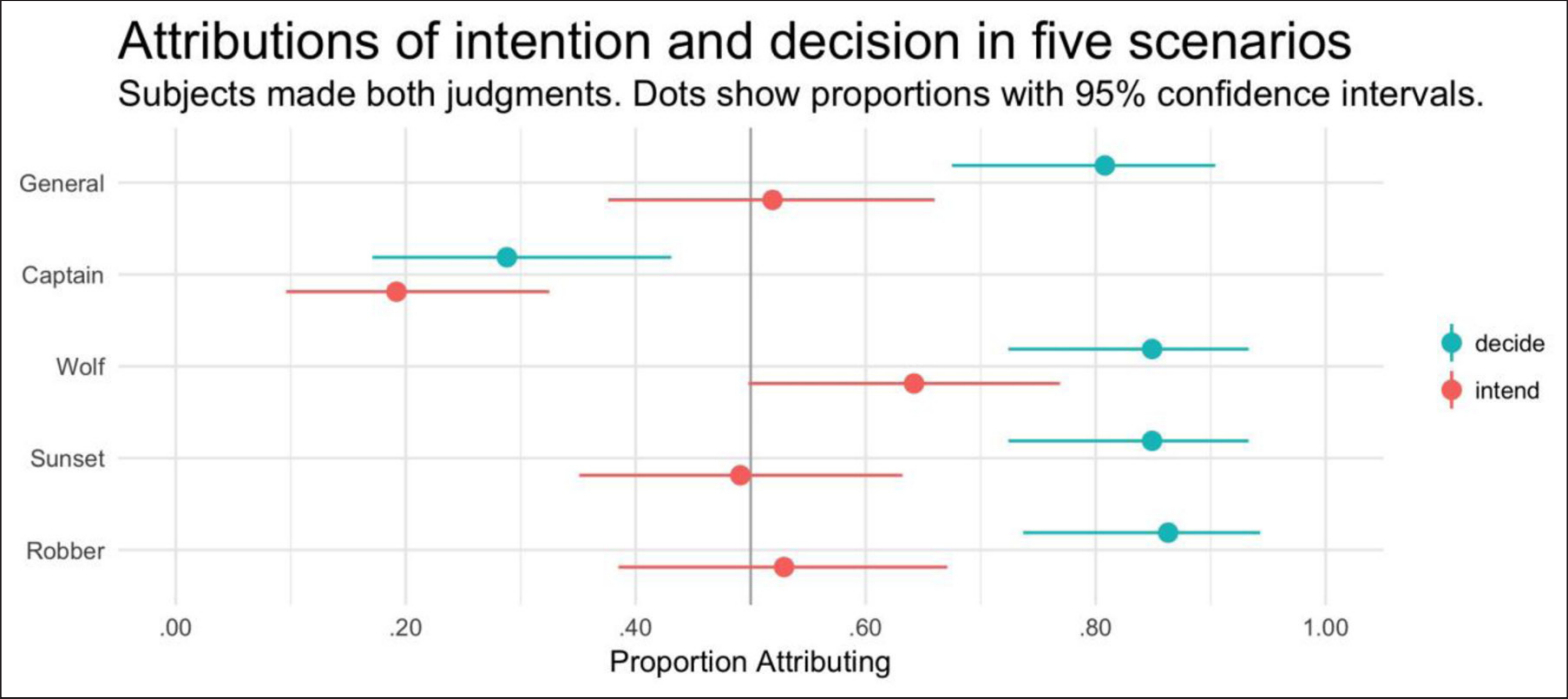
Experiment 5. Proportion of participants attributing intent, decision, and responsibility (within-subjects). Error bars show 95% bootstrapped confidence intervals.

The analysis revealed a main effect of judgment type (see Table 13), with participants significantly more likely to attribute decision than intention. Although the difference did not reach significance for each scenario individually (see Figure 5 and Table 14), the numerical difference was always in the same direction. Overall, 47.5% of participants attributed intention, which did not differ from chance, and 73.2% attributed decision, which significantly exceeded chance (see Tables 15 and 16).

**Table 13:** Experiment 5. Generalized linear mixed model’s fixed effects.

Term	estimate	std. error	z	p

(Intercept)	–0.793	0.685	–1.158	.247
Decide	1.683	0.281	5.989	<.001
Female	0.221	0.280	0.791	.429
Age	0.013	0.012	1.024	.306

**Table 14:** Experiment 5. Descriptive statistics, binomial tests, and effect sizes for the test items across all conditions.

Condition	Judgment	n	k	prop	conf.low	conf.high	test.prop	p	h

General	intend	52	27	.519	.376	.660	.5	.890	0.038
General	decide	52	42	.808	.675	.904	.5	<.001	0.663
Captain	intend	52	10	.192	.096	.325	.5	<.001	–0.663
Captain	decide	52	15	.288	.171	.431	.5	.003	–0.437
Wolf	intend	53	34	.642	.498	.769	.5	.053	0.287
Wolf	decide	53	45	.849	.724	.933	.5	<.001	0.773
Sunset	intend	53	26	.491	.351	.632	.5	1	–0.019
Sunset	decide	53	45	.849	.724	.933	.5	<.001	0.773
Robber	intend	51	27	.529	.385	.671	.5	.780	0.059
Robber	decide	51	44	.863	.737	.943	.5	<.001	0.812

**Table 15:** Experiment 5. Descriptive statistics, binomial tests, and effect sizes for the test items across all conditions.

Judgment	n	k	prop	95 CI low	95 CI high	test value	p	h

intend	261	124	.475	.413	.538	.5	.458	–0.050
decide	261	191	.732	.674	.785	.5	<.001	0.482

**Table 16:** Experiment 5. Counts of participants who made the four possible combinations of intent and decision: deny (= 0) or attribute (= 1).

Intend	Decide	Frequency

0	0	58
1	0	12
0	1	79
1	1	112

### 7.3. Discussion

This study attempted to generalize the earlier studies’ principal finding of interest, namely, that decision attributions exceed intent attributions. Across five very different scenarios we find that decision judgments tend to exceed intention judgments. Whereas one concern with extant evidence against the equivalence thesis is that it only concerns a single case (McGuire, 2016), our findings concern a broad range of very different cases. The fact that that decision judgments outpace intention judgments across a range of cases provides strong evidence against the equivalence thesis.

## 8. General Discussion

Is “deciding” to do something synonymous with “forming an intention” to do it? The consensus view that these phrases are synonymous has recently been challenged by the discovery of a new counterexample (McGuire 2016). While this constitutes the promising foundation for a new research program, the initial discovery should also be replicated and scrutinized before its philosophical significance can be fully understood. The main contribution of this paper has been to candidly assess these matters.

To that end, Experiment 1 replicated and extended the initial discovery by confirming that the same person will ascribe a decision without ascribing intent. We then considered one concern with Experiment 1, that perhaps participants were ascribing decision because they believed that he decided to start the program and merely *knew* that this would cause environmental harm. The findings from Experiment 2 show that the vast majority of participants preferred the option that the chairman decided to start the program and knew this would harm the environment, as opposed to the option that the chairman decided to start the program and decided to harm the environment. However one concern with Experiment 2 is that while participants were picking what they took to be the best description, they may have thought that the protagonist both decided and intended, but one statement better described the situation.

To address this concern, Experiment 3 asked participants to select all the descriptions that applied, rather than just choosing the response they find best. This study supports the original finding that one can decide to do something without forming an intention to do it. When asked to select all descriptions that apply, attributions of deciding still came apart from attributions of intending. A further concern one might have is that while the chairman case seems to be a typical “decision-making” context, it does not describe a stereotypical “intention-making” context. To address this concern, Experiment 4 gave participants the opportunity to rate attributions for both starting the program and increasing emissions. This study yielded mixed results, neither clearly supporting nor clearly undermining the initial finding. On the one hand, decision attribution was significantly higher than intent attribution. However, against the original finding, this difference was not significant in a full model controlling for other factors, overall participants tended to deny both decision and intent, and the proportion of participants who attributed decision but denied intent was numerically below chance rates.

A final question was whether previous findings are due to incidental features of testing a single scenario. To address this, Experiment 5 tested five different scenarios to see whether the results would generalize, and found, in fact, they do. Across all five cases, attributions of deciding were higher than attributions of intending. Together, these findings point to a robust tendency to attribute deciding without intending. These data uphold and extend the initial discovery and together provide a strong empirical foundation for the semantic view that, ordinarily understood, deciding does not entail intending.

## Data Accessibility Statement

All stimuli and data are available through an Open Science Foundation project (osf.io/m94zg/).

## Additional File

The additional file for this article can be found as follows:

10.5334/joc.101.s1 Appendix. Experiment 5 Materials.

## Footnotes

Throughout we will speak of “deciding” and “intending” where our focus is on the connection between deciding and forming an intention. “Intending” will sometimes be used as shorthand for “forming an intention”. “Intending” should not be read as “intending to X’. While there is a semantic connection between “forming an intention to X” and “intending to X”, these expressions are not synonymous (Mele 1992, 2003). And the equivalence thesis concerns only the semantic connection between “deciding” and “forming an intention”.
