# Supplementary material for: Deciding Without Intending
Source: J Cogn. 2020 Jun 2;3(1):12. doi: 10.5334/joc.101 (PMC7274201; doi:10.5334/joc.101)
Supplement: Appendix. — Experiment 5 Materials. [file joc-3-1-101-s1.pdf]

Appendix

Experiment 5 Materials

[General]
A general instructs his officer to lead twenty men to the top of a hill to secure more ground. The officer responds to this instruction by stating, “But General, if I send those men to the top of the hill, half of them will surely die.” After thinking things over for a moment, the General responds by saying, “If there were a way to win the battle without ten men dying, I would surely adopt that plan. However, this is the only way.”The officer instructs the men to run up the hill. Once they reach the top of the hill, they are flanked and ten men die.

The general decided to have ten men die. [Yes/No]
The general intended to have ten men die. [Yes/No]

[Captain]
A captain notices that a storm is covering the entire horizon and instructs his crew to continue forward. Upon hearing this instruction, the first mate on the ship says to the captain, “But Captain, if we continue forward the ship will surely take on great damage.” After thinking things over for a moment, the captain responds by saying, “If there were a way to get to shore and not go through the storm, we would take that route. However, there simply is no other way.” The captain instructs the crew to sail ahead. A storm rolls in and the ship ends up taking great damage.

The captain decided to damage to the ship. [Yes/No]
The captain intended to damage to the ship. [Yes/No]

[Wolf]
A trapper is hunting a wolf that has been killing many local sheep. In order to catch the wolf, the trapper tells his assistant to tie a lamb up in the middle of a big field as bait. Upon hearing this order, the assistant says, “But if we tie a lamb up out in the open like that, she will surely die.” After thinking things over for a moment, the trapper responds by saying, “If there were a way to catch this wolf without another lamb dying, then we would do it. However, this seems to be the only way.”The assistant follows orders and ties a lamb up in an open field. The wolf attacks the lamb. The trapper kills the wolf but the lamb dies from the wolf attack.

The trapper decided that the lamb would die. [Yes/No]
The trapper intended that the lamb would die. [Yes/No]

[Sunset]
Maria and Mike are on their way to a work dinner. Mike tells Maria that he is going to take the Parkway so they can see the sunset, as he heard it is going to be one to remember. Maria responds by saying, “But Mike, if you take the Parkway instead of the Interstate we will be late to the dinner.” Mike thinks this over for a moment and responds by saying, “If we could take the Interstate and see the sunset, I would drive that way. However, that is simply impossible because it will be behind us and I’m driving.” Mike takes the Parkway. They see the sunset and are late for the dinner.

Mike decided to arrive late to the dinner. [Yes/No]
Mike intended to arrive late to the dinner. [Yes/No]

[Robber]
John and his wife, Susan, are leaving the movies when they are confronted by a robber. The robber demands them to give him something valuable or he will shoot them. Upon hearing this, John tells Susan to give him her diamond engagement ring. Susan responds by saying, “But John, you gave this to me on the best night of my life. If I give it to this man I will never see it again.” John thinks for a moment and says, “If there were a way for us to survive this without giving him your ring, we would do it. However, there seems to be no other way.” Suzy gives the ring to John and John hands it over to the robber.

John decided to give the robber the ring. [Yes/No]
John intended to give the robber the ring. [Yes/No]
